# The impact of food assistance on weight gain and disease progression among HIV-infected individuals accessing AIDS care and treatment services in Uganda

**DOI:** 10.1186/1471-2458-10-316

**Published:** 2010-06-07

**Authors:** Rahul Rawat, Suneetha Kadiyala, Paul E McNamara

**Affiliations:** 1 Poverty, Health, and Nutrtition Division, International Food Policy Research Institute, 2033 K Street, Washington D.C. 20006, USA; 2 Concern Worldwide, 52-55 Lower Camden Street, Dublin 2, Ireland; 3 Department of Agricultural and Consumer Economics and Division of Nutritional Sciences, University of Illinois at Urbana-Champaign, 341 Mumford Hall, 1301 W. Gregory Drive, Urbana, Illinois, 61801, USA

## Abstract

**Background:**

The evidence evaluating the benefits of programmatic nutrition interventions to HIV-infected individuals in developing countries, where there is a large overlap between HIV prevalence and malnutrition, is limited. This study evaluates the impact of food assistance (FA) on change in weight and disease progression as measured by WHO staging.

**Methods:**

We utilize program data from The AIDS Support Organization (TASO) in Uganda to compare outcomes among FA recipients to a control group, using propensity score matching (PSM) methods among 14,481 HIV-infected TASO clients.

**Results:**

FA resulted in a significant mean weight gain of 0.36 kg over one year period. This impact was conditional on anti-retroviral therapy (ART) receipt and disease stage at baseline. FA resulted in mean weight gain of 0.36 kg among individuals not receiving ART compared to their matched controls. HIV-infected individuals receiving FA with baseline WHO stage II and III had a significant weight gain (0.26 kg and 0.2 kg respectively) compared to their matched controls. Individuals with the most advanced disease at baseline (WHO stage IV) had the highest weight gain of 1.9 kg. The impact on disease progression was minimal. Individuals receiving FA were 2 percentage points less likely to progress by one or more WHO stage compared to their matched controls. There were no significant impacts on either outcome among individuals receiving ART.

**Conclusions:**

Given the widespread overlap of HIV and malnutrition in sub-Saharan Africa, FA programs have the potential to improve weight and delay disease progression, especially among HIV-infected individuals not yet on ART. Additional well designed prospective studies evaluating the impact of FA are urgently needed.

## Background

The interactions between food insecurity, malnutrition, and HIV/AIDS are well established [1,2]. Earlier studies from developed and developing countries consistently show that malnutrition, as assessed by low body mass index (BMI) or mid-upper arm circumference (MUAC), is a strong independent predictor of mortality among people living with HIV (PLHIV) [3-6]. Indeed, despite increasing access to anti-retroviral therapy (ART) in resource limited settings, recent evidence has emerged establishing preexisting malnutrition as an independent risk factor of mortality among patients initiating ART [7-9]. Combined, this evidence has resulted in increased funding to HIV care and treatment providers to provide nutrition support to adults and children with HIV, especially those who are malnourished or have advanced HIV disease. The hypothesized benefits of providing nutrition support to HIV-infected individuals include: i) improved nutritional status, ii) improved health status, iii) slowed disease progression, iv) improved food security, v) improved quality of life, and vi) improved ART adherence and probability of survival.

The evidence base evaluating the benefits of nutrition interventions in developing countries, where there is a large overlap between HIV prevalence and malnutrition is limited to efficacy studies of micronutrient supplementation and more recently, specialized ready-to-use foods. The benefits of food assistance (FA) programs, which remain the most widespread form of nutrition support to HIV-infected individuals in sub-Saharan Africa, are mostly speculative and have not been rigorously evaluated.

Two recent reviews on the effect of macronutrient supplementation (with or without nutritional counseling) on various clinical outcomes of people living with HIV offer inconclusive evidence of the impact on weight gain or CD4 count in developed countries [10,11]. Of the studies reviewed, only two studies were available from resource constrained settings with the bulk of evidence provided by nine small clinical trials, all of them in the United States or Europe, where the preexisting nutrition status of people living with HIV is far superior to those in the developing countries.

A challenge to generating rigorous evidence of the impact FA programs on PLHIVs in countries where there is widespread co-existence of malnutrition, food insecurity, and HIV is the important ethical considerations in conducting experimental studies with randomized control groups that receive no intervention. However, data from program settings is often available, and if rigorous methodologies are applied, this data can be utilized to build a plausibility argument of the potential impact of FA programs.

We capitalize on the availability of routine program data from The AIDS Support Organization (TASO), the largest indigenous HIV care and treatment service provider in Uganda serving over 190,000 clients since its inception, to evaluate the effectiveness of FA provided (by WFP and USAID in the form of a monthly household food basket) to HIV-infected TASO clients on weight gain, and advancement in WHO disease stage. Given the absence of a randomized control group in this program setting, we compared HIV-positive individuals who received FA with a matched control group with similar observed characteristics, using propensity score matching (PSM).

During the years 2002-7, TASO clients were provided two different types of household food rations. Based on geographic targeting of the food distributor and household size (with a upper household size limit of 6), TASO clients were provided either a: 1) household food ration by the World Food Programme (WFP) comprising corn soy blended flour, vegetable oil, pulses and maize meal or rice, or 2) a household food ration by USAID comprising corn soy blended flour and vegetable oil. Food rations were distributed monthly, barring any pipeline breakdowns. These household rations and their mode of delivery represent standard food commodities and a distribution model provided to households as part of food aid programs throughout sub-Saharan Africa. TASO clients were provided food assistance for varying periods of 12-24 months based on resources, but we restricted our analysis to available client record information closest to the 12-month mark.

## Methods

### Data

The data set utilized in this analysis builds upon TASO's electronic monitoring data system. This information system includes computerized information from the patient's intake registration form, medical visit summaries, counseling visit summaries, as well as information on ART initiation and other drug use. We conducted a secondary data analysis of the records, and we analyzed patient information for the time period 2002-2007. For each TASO client, we retrieved all of their medical and counseling visit files as well as their registration intake survey information. This permitted the analysis to consider social and economic information such as occupation and educational attainment, essential for PSM, along with health information including WHO staging and weight.

The study question of this paper focuses on changes over 12 months for patients and how the receipt of FA affects health outcomes measured in terms of change in WHO staging and weight of patients receiving TASO care and treatment services. Thus, from the patient database, we pulled patient records from the first medical visit and obtained baseline weight and WHO staging information. Then we searched the database and pulled the record closest to 365 days after the first medical visit. We dropped patients with no recorded visit in the time period of between 160 days and 560 days from the first visit from the analysis.

The TASO data we utilize is generated through the program's management information system (a paper system of charts and records) and then transferred to a set of electronic databases. By the end of 2007, TASO had registered 195,676 program participants, from 10 TASO clinic sites across Uganda. Data cleaning involved dropping patients with incomplete identification numbers (necessary to link across the patient records databases) or those with no program involvement in the 2002-2007 time period. Additionally, patients were dropped if they did not have a clinic visit in the 160 to 560 day window, or if they had missing weight or WHO staging information. The final number of complete observations with the necessary weight and WHO records were 14, 481.

There are unique challenges to using data from program settings. In the case of the TASO program database, there are three main challenges. First, as seen above, not all TASO clients have information recorded consistently in the TASO database system, a pervasive challenge among large AIDS care and treatment providers in general. Second, the available data is limited to variables that are collected for purposes of program monitoring, and do not include a full array of variables that would ordinarily be collected for research purposes. Third, receipt of FA is not random, and may correlate with several confounding characteristics which themselves could influence the outcomes of interest. FA eligibility in TASO's program is determined by a poverty assessment tool, which includes several socio-economic criteria. The main methodological challenge to the non-random receipt of FA is that failure to control for this characteristic may generate biased results of the impact of FA on the outcomes of interest. The key question of interest therefore is determining how HIV-infected individuals receiving FA would have fared had they not received food. This paper seeks to provide insight into this research question using propensity score matching (PSM) techniques.

The poverty assessment tool to determine food assistance eligibility from WFP covers the following domains: household composition, ownership of valuable assets, employment status and income, housing characteristics, experience of food insecurity, and access to certain services. The poverty assessment tool to determine food assistance eligibility for USAD Title II food aid is less comprehensive. It elicits descriptive information on household assets, household composition (mainly number of dependents), adequacy of income to meet basic needs and experience of food insecurity. The tools were administered to TASO clients and scored to determine eligibility to food assistance.

In addition to the differing tools, during the study period of five years (2002-7), the cut-off score to determine eligibility varied, based in part on the availability of program resources in different geographic locations where food assistance programs operated. Since food aid programming in the context of HIV was relatively new at the beginning of the decade, the tools were also revised. Because of these variations in the tools and in determining eligibility across TASO program sites, relying on the poverty assessment tool alone would have provided erroneous impact estimates.

We matched individuals using propensity score matching methods, using more detailed responses to the domains captured in the poverty assessment tool as well as additional variables not captured in the poverty assessment tool. These variables were captured from patient records that are part of the TASO intake registry. This allows for a more robust matching technique that goes beyond the limited set of variables and response options in the poverty assessment tool.

### Propensity Score Matching

We estimated the impact of food assistance using propensity score matching with difference-in-difference (DID) estimates. This statistical procedure compares the change over time in the outcomes of interest for food assistance TASO clients with the change over time to matched comparison TASO clients.

To understand the true effect of FA, we need to estimate a) the outcomes of interest among those receiving FA and b) the outcomes of interest on the same individual under the same conditions, but without receipt of FA. Since the latter is unobservable, we need to construct a proxy counterfactual for the missing data. In order to do this, information on each FA recipient is matched to a sample of similar non-FA recipients for the same two periods [12]. PSM allows us to match each FA recipient with *similar *non-FA recipients, and use the outcome of the non-FA recipients as a proxy for the outcome of the FA recipients if they had not received FA [13].

There may be something about FA recipients that makes them different from the general set of households. For example, if an individual with a higher number of dependents is more likely to receive FA, we would want to consider the number of dependents when matching individuals. Thus, matching entails identifying a set of characteristics that are associated with an increased propensity to receive FA and likely to influence the outcome [14].

PSM involves two steps. First, obtaining propensity scores involves estimating a probit or a logit model that predicts the probability of each PLHIV receiving FA as a function of baseline characteristics using the sample of FA recipients and non-recipients. Second, for each FA recipient, matching involves finding a non- FA recipient with the closest estimated propensity score. The matching estimator was implemented using kernel matching.

This study estimates the effect of FA on outcomes on FA recipients. This is known in the literature as the "average treatment effect on the treated" (ATT). The ATT is obtained by comparing the change in means of the outcome of interest between the matched FA and non-FA recipient. It is plausible that unobservable characteristics that are uncorrelated to observables may still bias the impact estimations after PSM. In order to further improve the impact estimations, we employ difference-in-difference (DID) estimation. DID is the pre and post difference between treatment and control groups. DID estimates are known to be less subject to selection bias because they remove the effect of any unobserved time-invariant differences between the treatment and comparison groups [15]. We estimated the overall and conditional effects of FA on changes in weight and disease progression by one or more WHO stages.

### Variable Description

The main treatment variable is receipt of FA in the form of a household food basket ration. The primary outcome variables of interest are: a) change in weight in kilograms, and b) advancement in WHO disease staging by one or more stages. We estimated a logit to compute the propensity score. The log odds of receiving FA was estimated by a set of independent variables at baseline including sex, age, number of dependents, education, religion, occupation, marital status, time in days from the initial visit to the visit closest to one year from the initial visit, and distance to a TASO medical center. We further included a set of TASO site dummy variables to control for community level characteristics.

### Ethical Review

The ethics review boards of TASO, the Uganda National Council on Science and Technology (UNCST) and IFPRI approved the study protocol.

## Results

### Descriptive Statistics

Our final sample for analysis included 14, 481 observations, twenty-three percent of whom reported receiving food assistance (Table 1). Included in this table are descriptive characteristics of select variables of interest. Here, we discern some significant differences in baseline characteristics of FA recipients and non-recipients. The overall mean weight gain over a period of one year in this population was 0.95 kg; FA recipients had a mean weight gain of 1.2 kg, compared to a mean weight gain of 0.85 kg among non-recipients. Overall, 24% of the cohort experienced disease progression by one or more WHO stages; this was slightly higher among non-FA recipients (25%) compared to FA recipients (23%). These changes in our outcomes of interest over a period of one year are raw values, not based on PSM methods.

**Table 1:** Baseline characteristics TASO clients receiving and not receiving food assistance

	Recipients of Food Assistance (n = 3370)	Non Recipients of Food Assistance (n = 11,111)
**Age (yrs)**	38.3	39.0

**Sex (Female)**	2,623 (77.8%)	7,814 (70.3%)

**Receipt of ART**	556 (16.5%)	1,155 (10.4%)

**Baseline Weight (kgs)**	53.6	53.5

**Baseline WHO Status**		
Stage I	334 (9.9%)	1,546 (13.9%)
Stage II	2,358 (70.0%)	7,502 (67.5%)
Stage III	619 (18.4%)	1,886 (17.0%)
Stage IV	59 (1.8%)	177 (1.59%)

**Education Level**		
Primary	1,192 (59.1%)	6,586 (59.3%)
Secondary	642 (19.1%)	1,960 (17.6%)
Other/None	736 (21.8%)	2,565 (23.1)

**Marriage Status**		
Married/Cohabiting	1,439 (42.7%)	5,274 (47.5%)
Divorced/Separated	544 (16.1%)	1,721 (15.5%)
Never Married	96 (2.9%)	383 (3.5%)
Widowed	1,235 (36.7%)	3,448 (31.0%)
Other	56 (1.7%)	285 (2.6%)

**Occupation**		
Casual Laborer	270 (8.0%)	826 (7.4%)
Paid Employee	157 (4.7%)	649 (5.8%)
Peasant Farmer	1,312 (38.9%)	5,529 (49.8%)
Vendor	295 (8.8%)	893 (8.0%)
Other	1,336 (39.6%)	3,214 (28.9%)

**Distance to TASO Center**		
<10 kms	1,527 (45.3%)	2,972 (26.8%)
10-20 kms	623 (18.5%)	1,716 (15.4)
>20 kms	1,220 (36.2%)	6,423 (57.8%)

### Propensity Score Matching Results

**Figure 1:**
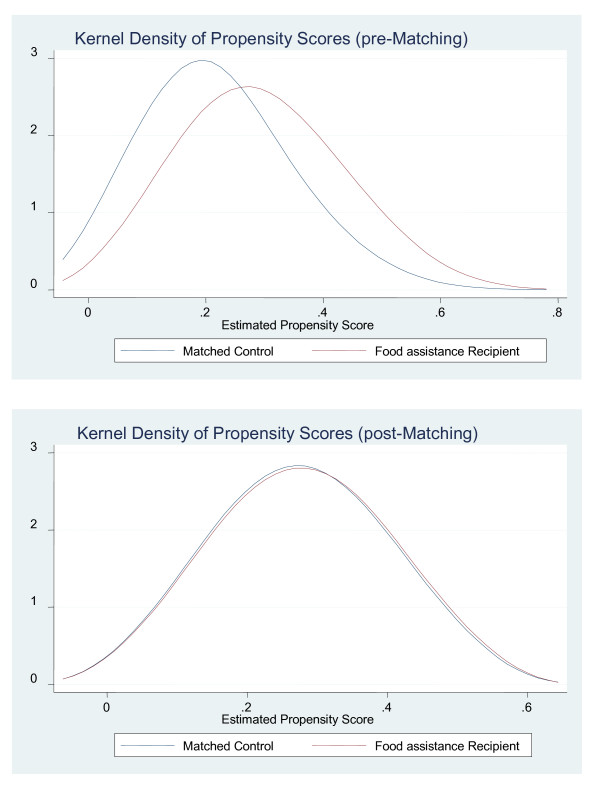
**Kernel density plots before and after matching**.

After estimating the propensity score, we generated a sample of matched FA recipients and non- FA recipients. FA recipients with an estimated propensity score above the maximum or below the minimum propensity score for the comparison group were treated as not having ''common support" in the comparison group and so were dropped from the matched sample [12,16]. Figure 1 shows the kernel density plots of the estimated propensity scores before and after matching. Here, we observe negligible difference in the mean propensity scores and their distribution of the two groups after kernel matching, indicating that we were successful in our matching.

### Impact of Food Assistance on Weight Gain

Overall, we observe that FA resulted in a statistically significant mean weight gain of 0.36 kg over the one year period (Table 2). The disaggregated analyses show that the impact of FA was conditional on both the receipt of ART, and baseline HIV disease status as measured by WHO stage. FA resulted in a statistically significant weight gain of 0.36 kg during the one year study period among PLHIVs not receiving ART compared with their matched controls (individuals neither receiving FA nor ART). PLHIVs receiving ART and FA did not have a significant weight gain compared to their matched controls (individuals receiving ART, but not FA). There was no observed impact of FA among PLHIVs who were in the early stages of HIV, as characterized by WHO stage I at baseline (Table 2). We discern a positive and statistically significant impact of FA among individuals with a more advanced disease stage at baseline (WHO stage II onwards). PLHIVs receiving FA at baseline WHO stage II and III had a significant weight gain (0.26 kg and 0.2 kg respectively) compared to their matched controls with the same baseline WHO stage who did not received FA. Those with the most advanced disease stage at baseline (WHO stage IV, often characterized by severe wasting), had the highest weight gain point estimate over the one year period (1.9 kg).

**Table 2:** Average treatment effect (ATT) of food assistance on change in weight of people living with HIV

Change in Weight	Food assistance Recipients (n)	Matched Controls (n)	**ATT**^**a **^**(absolute value of t-statistic)**
Overall	3329	10685	0.36 (3.46)**

***Conditional estimates***			

Without ART	2783	9661	0.36 (2.40)*

With ART	546	1120	0.19 (0.43)

Baseline WHO stage 1	327	1479	-0.2 (0.55)

Baseline WHO stage 2	2329	7318	0.26 (2.3)*

Baseline WHO stage 3	615	1807	0.2 (1.8)^+^

Baseline WHO stage 4	58	129	1.9 (1.9)^+^

^a ^Absolute value of t-statistics on ATT, in parentheses, are based on bootstrapped standard errors

** significant at 1% ; *significant at 5% ; + significant at 10%

### Impact of Food Assistance on WHO Disease Staging

While FA significantly slowed disease progression, as measured by advancement by one or more WHO stage (Table 3), the effect size of this impact is minimal. Over the course of one year, PLHIVs who received FA were two percentage points less likely to progress by one WHO disease stage or more, compared to their matched controls. Among those who did not receive ART, individuals who received FA were three percentage points less likely to worsen than their matched controls (individuals neither receiving ART, nor FA). We found no impact of FA among individuals who received ART.

**Table 3:** Average treatment effect (ATT) of food assistance on advancement in WHO stage by one or more WHO stage

Change in HIV WHO stage	Number of treated	Number of matched controls	**ATT**^**a **^**(absolute value of t-statistic)**
**Overall**	3329	10805	-0.02 (2.14)*

***Conditional estimates***			

Without ART	2783	9661	-0.03 (3.43)**

With ART	546	1120	0.025 (0.95)

^a ^Absolute value of t-statistics on ATT, in parentheses, are based on bootstrapped standard errors

** significant at 1% ; *significant at 5%

## Discussion

International organizations such as the World Health Organization (WHO), the Joint United Nations Programme on HIV/AIDS (UNAIDS), World Food Program (WFP), Food and Agriculture Organization (FAO) and The United States President's Emergency Plan for AIDS Relief (PEPFAR) have recommended integration of food assistance into AIDS care and treatment programs [17,18]. Several of these organizations have tailored interventions to specifically address malnutrition and food insecurity in areas with high prevalence of HIV, particularly in sub-Saharan Africa. In 2007, PEPFAR integrated food assistance interventions as an integral part of AIDS care and treatment services. Health care providers and NGOs involved in HIV care and treatment are now increasingly utilizing targeted food assistance to improve nutritional and clinical outcome of their clientele. Despite this growing recognition and activity in integrating targeted food assistance to PLHIVs in the context of HIV, there have been few rigorous studies to evaluate the health and nutritional impact of these programs.

While food assistance programs to HIV affected households are widespread, studies evaluating the impact of these programs on people living with HIV and with pre-existing food and nutrition insecurity are just beginning to emerge. Given the ethical and programmatic challenges of randomizing food assistance in chronically food insecure and high HIV prevalence contexts, most studies currently being carried out employ quasi experimental designs with control groups or randomize participants to two different types of food supplementation regimens. At present, there are only 2 published studies that evaluate the impact of macronutrient supplementation to HIV infected individuals in a resource-constrained setting. In a quasi-experimental study in Zambia, food supplementation was associated with better adherence to ART, after adjustment for sex, age, and baseline CD4 count, WHO stage, and hemoglobin [19]. In this study however, there was no significant effect of food supplementation on weight gain or disease progression. In a randomized controlled trial from Malawi [20] comparing supplementary feeding with a ready-to-use fortified spread compared to corn-soy blended flour with a similar energy composition, patients receiving fortified spread had a greater increase in BMI and fat-free body mass than those receiving corn-soy blend, but there were no significant observed differences in markers of disease progression, quality of life, or adherence to ART between the two groups.

Our study contributes to the literature on understanding the impact of food assistance on HIV-infected individuals. The commodities provided are the most commonly available supplementary foods in food aid programs in sub-Saharan Africa. Using data from a large programmatic setting, we observe an overall positive impact of food assistance on weight gain, and a minimal impact on delaying disease progression as measured by WHO staging. The impact on weight gain was greatest among individuals with more the most advanced disease stage at baseline, where we observe an increase of almost 2 kg, which was significant at the 10% level, despite there being very few individuals categorized as WHO Stage IV at baseline. It is plausible that there is selective attrition of individuals with more advanced stage, especially in the early years in the panel when ART was not as widely accessible. A part of the attrition in the monitoring database (and not necessarily TASO services) could also be due to the expansion of TASO's home based care and treatment programs and consequently less attention to entering data into monitoring databases. The observation of a greater impact of food assistance among those with more advanced disease at baseline is in line with our *a priori *belief --advanced HIV disease is characterized by severe weight loss and wasting, and the potential to benefit is greatest among these individuals. Our results also show that weight gain is observed among individuals with less advanced disease, though the point estimates of impact are lower. While the impacts of food assistance on disease progression are small in magnitude, these results are important nonetheless, considering our inability to carefully control data collection or the compliance and follow-up of TASO clients in this large, overburdened, real-life program setting where care and treatment service provision are the primary goals. We contend that in a carefully controlled study, the impacts of food assistance would likely be significantly greater on our outcomes of interest.

It is important to highlight that the lack of observed impact among the subset of individuals receiving ART should be interpreted with caution. We do not in any way suggest, nor should it be inferred, that nutrition support to individuals receiving ART is of no benefit. The current consensus is clear; malnutrition and food insecurity have emerged as major barriers to the effectiveness of ART programs in sub-Saharan Africa. There are several factors that deserve discussion. First, our data does not allow us to determine when during the course of taking ART, food assistance was provided. Ideally, we would have designed a prospective study that allowed us to examine the impact of food assistance immediately upon starting ART. We have no ability to determine when ART was initiated, or disaggregate our analysis by time on ART. Second, availability of ART was severely limited, especially in the early part of the decade, and PLHIVs with only the most advanced disease stage were given ART. Related to this, we have no measure of compliance or duration of ART. It is therefore not surprising that we do not find an impact of food assistance on advancement of HIV by one or more WHO stages among those on ART. Now, with the wider availability of ART and revision of guidelines for their initiation, prospective studies are critically needed to understand the impact of FA among those initiating ART. Lastly, it is possible that food assistance may have had an impact on other important outcomes such as ART adherence and quality of life, the study of which is beyond the scope of this paper.

Additionally, there may be other benefits of food assistance that our data was unable to examine. For example, lack of availability of food is widely cited to be a threat to ART adherence, but our data does not allow us to examine the impact of food assistance on this important outcome. Although this paper does not investigate ART adherence as an outcome, the role of food assistance programs in enabling successful ART programming cannot be ruled out. At the household level, other potential benefits of food assistance might include improved household food security, nutritional status of children, providing a cushion for other household expenses such as medication, school fees etc.

Like other health data systems in sub-Saharan Africa, the TASO electronic data system does face the challenges of completeness and accuracy of data elements (see for example, [21]). Despite the short-comings noted above related to missing data elements for the critical variables of weight and WHO staging, among others, we contend that the TASO data allow plausible inferences that are central to the understanding of the role of food support and nutrition to the health outcomes of people in treatment for HIV and AIDS. As others have noted, many important questions in the area of evidenced-based nutrition or health policy cannot be answered easily with randomized controlled trials [22,23]. The TASO data has the attribute of generalizability because it reflects the impacts of food support, using a widely implemented program model, on health outcomes for a person not in a carefully regulated and monitored clinical trial, but among individuals receiving the day-to-day standard of care of an indigenous NGO. Additionally, the TASO data shed light on the role of food support in HIV and AIDS care, a phenomenon which is extremely difficult to imagine designing a randomized clinical trial because of ethical issues, but where observational data may allow inferences to be constructed given naturally (quasi-experimental) occurring variation. For these reasons, the TASO data, despite their limitations, offer a unique source of information concerning food support and HIV and AIDS health outcomes.

## Conclusions

Despite the limitations of our study, through construction of a plausible control group and rigorous analysis of program data, our finding of positive impacts of nutritional support to HIV-infected individuals in a resource-limited setting makes an important contribution to the evidence of the benefit of food assistance programs. These findings are highly relevant to program implementers. Further carefully designed prospective studies evaluating the impact of nutrition interventions employing experimental designs (where feasible) or thoughtful and rigorous quasi-experimental designs, to improve health, nutrition and quality of life outcomes among HIV-infected individuals are critically needed. Donor agencies providing food assistance integrated with HIV care and treatment programs should prioritize the need for such research.

## Competing interests

The authors declare that they have no competing interests.

## Authors' contributions

SK and PM originated the study. PM constructed the database. SK conducted the data analysis, and together with RR interpreted the data. RR and SK wrote the paper, with inputs from PM. All authors read and approved the final manuscript.

## Pre-publication history

The pre-publication history for this paper can be accessed here:

http://www.biomedcentral.com/1471-2458/10/316/prepub

## References

[B1] Gillespie S Kadiyala S HIV/AIDS and Food and Nutrition Security: From Evidence to Action 2005 Washington DC, International Food Policy Research Institute Ref Type: Report

[B2] Anema A Vogenthaler N Frongillo EA Kadiyala S Weiser SD Food insecurity and HIV/AIDS: current knowledge, gaps, and research priorities Curr HIV/AIDS Rep 2009 6 224 231 10.1007/s11904-009-0030-z 19849966 PMC5917641

[B3] World Health Organization Consultation on Nutrition and HIV/AIDS in Africa: Evidence, lessons, and recommendations for action 2005 Geneva Ref Type: Report

[B4] Villamor E Misegades L Fataki MR Mbise RL Fawzi WW Child mortality in relation to HIV infection, nutritional status, and socio-economic background International Journal of Epidemiology 2005 34 61 68 10.1093/ije/dyh378 15649965

[B5] van der Sande MAB van der Loeff MFS Bennett RC Dowling M Aveika AA Togun TO Sabally S Jeffries D Adegbola RA Incidence of tuberculosis and survival after its diagnosis in patients infected with HIV-1 and HIV-2 AIDS 2004 18 1933 1941 10.1097/00002030-200409240-00009 15353979

[B6] Zvandasara P Hargrove JW Ntozini R Chidawanyika H Mutasa K Iliff PJ Moulton LH Mzengeza F Malaba LC Mortality and morbidity among postpartum HIV-positive and HIV-negative women in Zimbabwe: risk factors, causes, and impact of single-dose postpartum vitamin A supplementation J Acquir Immune Defic Syndr 2006 43 107 116 10.1097/01.qai.0000229015.77569.c7 16885772

[B7] Johannessen A Naman E Ngowi BJ Sandvik L Matee MI Aglen HE Gundersen SG Bruun JN Predictors of mortality in HIV-infected patients starting antiretroviral therapy in a rural hospital in Tanzania BMC Infect Dis 2008 8 52 10.1186/1471-2334-8-52 18430196 PMC2364629

[B8] Zachariah R Fitzgerald M Massaquoi M Pasulani O Arnould L Makombe S Harries AD Risk factors for high early mortality in patients on antiretroviral treatment in a rural district of Malawi AIDS 2006 20 2355 2360 10.1097/QAD.0b013e32801086b0 17117022

[B9] Paton NI Sangeetha S Earnest A Bellamy R The impact of malnutrition on survival and the CD4 count response in HIV-infected patients starting antiretroviral therapy HIV Med 2006 7 323 330 10.1111/j.1468-1293.2006.00383.x 16945078

[B10] Koethe JR Chi BH Megazzini KM Heimburger DC Stringer JS Macronutrient supplementation for malnourished HIV-infected adults: a review of the evidence in resource-adequate and resource-constrained settings Clin Infect Dis 2009 49 787 798 10.1086/605285 19624276 PMC3092426

[B11] Mahlungulu S Grobler LA Visser ME Volmink J Nutritional interventions for reducing morbidity and mortality in people with HIV Cochrane Database Syst Rev 2007 CD004536 17636766 10.1002/14651858.CD004536.pub2

[B12] Heckman JJ Ichimura H Todd PE Matching as an econometric evaluation estimator: Evidence from evaluating a job training programme Review of Economic Studies 1997 64 605 654 10.2307/2971733

[B13] Rosenbaum PR Rubin DB The Central Role of the Propensity Score in Observational Studies for Causal Effects Biometrika 1983 70 41 55 10.1093/biomet/70.1.41

[B14] Becker S Ichino A Estimation of Average Treatment Effects Based on Propensity Scores The Stata Journal 2009 2 358 377

[B15] Heckman JJ Ichimura H Todd PE Matching as an econometric evaluation estimator: Evidence from evaluating a job training programme Review of Economic Studies 1997 64 605 654 10.2307/2971733

[B16] Smith JA Todd PE Does matching overcome LaLonde's critique of nonexperimental estimators? Journal of Econometrics 2005 125 305 353 10.1016/j.jeconom.2004.04.011

[B17] WFP, WHO & UNAIDS UNAIDS Policy Brief: HIV, Food Security, and Nutrition 2008 Ref Type: Report

[B18] PEPFAR Policy Guidance on the Use of Emergency Plan Funds to Address Food and Nutrition Needs 2006 Ref Type: Report

[B19] Cantrell RA Sinkala M Megazinni K Lawson-Marriott S Washington S Chi BH Tambatamba-Chapula B Levy J Stringer EM A pilot study of food supplementation to improve adherence to antiretroviral therapy among food-insecure adults in Lusaka, Zambia J Acquir Immune Defic Syndr 2008 49 190 195 10.1097/QAI.0b013e31818455d2 18769349 PMC3847664

[B20] Ndekha MJ van Oosterhout JJ Zijlstra EE Manary M Saloojee H Manary MJ Supplementary feeding with either ready-to-use fortified spread or corn-soy blend in wasted adults starting antiretroviral therapy in Malawi: randomised, investigator blinded, controlled trial BMJ 2009 338 b1867 10.1136/bmj.b1867 19465470 PMC2685879

[B21] Mate KS Bennett B Mphatswe W Barker P Rollins N Challenges for routine health system data management in a large public programme to prevent mother-to-child HIV transmission in South Africa PLoS One 2009 4 e5483 10.1371/journal.pone.0005483 19434234 PMC2677154

[B22] Habicht JP Victora CG Vaughan JP Evaluation designs for adequacy, plausibility and probability of public health programme performance and impact Int J Epidemiol 1999 28 10 18 10.1093/ije/28.1.10 10195658

[B23] Victora CG Habicht JP Bryce J Evidence-based public health: moving beyond randomized trials Am J Public Health 2004 94 400 405 10.2105/AJPH.94.3.400 14998803 PMC1448265

